# Stirring the Debate: How Mixing Influences Reproducibility and Efficiency in Synthetic Organic Chemistry

**DOI:** 10.1021/acscentsci.5c01825

**Published:** 2026-01-06

**Authors:** Jasper H. A. Schuurmans, Stefan D. A. Zondag, Arnab Chaudhuri, John van der Schaaf, Timothy Noël

**Affiliations:** † Flow Chemistry Group, Van’t Hoff Institute for Molecular Sciences (HIMS), 1234 Universiteit van Amsterdam (UvA), 1098 XH Amsterdam, The Netherlands; ‡ Department of Chemical Engineering and Chemistry, Sustainable Process Engineering, 3169 Eindhoven University of Technology (TU/e), 5612 AZ Eindhoven, The Netherlands; § Circular Chemical Engineering, 5211 Maastricht University, 6167 RD Geleen, The Netherlands

## Abstract

Mixing is essential in chemical processes, ensuring the proximity and interaction of reactants. Recent reports suggest stirring may minimally affect some solution-phase organic reactions, but this oversimplifies mixing’s complexity. We discuss its principles, relevance to organic synthesis, and practical considerations for reproducibility and safety. Even if some reactions seem agitation-insensitive, mixing remains crucial for reproducibility, scalability, and industrial applications.

## Graphical abstract

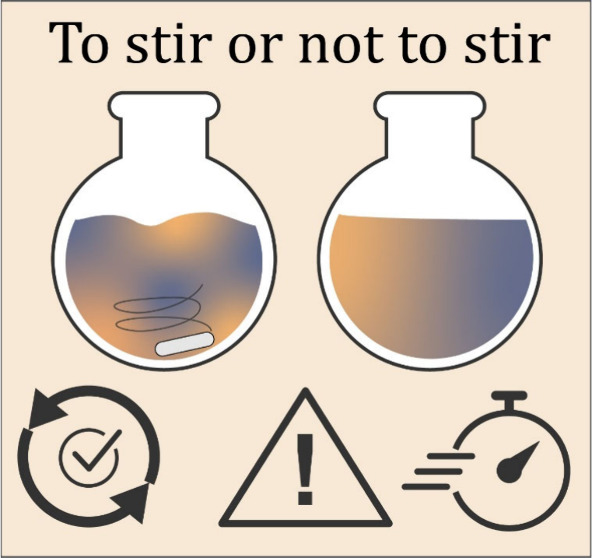

## 1 Introduction

Mixing and mixing efficiency have increasingly attracted the attention of organic chemists and materials scientists. Recent reports have presented contradictory conclusions about the role of stirring in chemical synthesis, revealing persistent misconceptions in the field. For example, one study reported that many organic reactions proceed with similar yields whether stirred or left standing, even suggesting that magnetic stir plates could be switched off to save energy and resources.^1^ In contrast, another study demonstrated that the positioning of vessels relative to a magnetic stirrer significantly affected nanoparticle synthesis, underscoring how mixing and equipment placement can critically influence the reaction outcome.^2^

Inspired by these seemingly opposing findings, we offer this *In Focus* article, providing an engineering perspective on the fundamentals of mixing and its implications for organic synthetic methodology. A classic example from laboratory practice highlights why mixing matters: acid dilutions are always performed by adding acid to water, never the reverse, to avoid dangerously high local concentrations and sudden temperature spikes. This simple case illustrates a fundamental goal of mixing: rapidly achieving homogeneity throughout a reaction vessel.

How, then, can it be that mixing is essential in some cases but appears irrelevant in others? The answer lies in the relative time scales of mixing and reaction. In the sections that follow, we address this question in detail by examining the underlying physical and chemical principles, aiming to clarify when mixing is indispensable and when it may truly be optional.

## 2 Mixing Principles: Diffusion vs Convection

The transport of chemical species in solution can be described through two fundamental mechanisms: diffusion and convection.

Diffusion arises from concentration gradients and is driven by the equalization of chemical potential, as described by Fick’s laws. It is an inherently slow process, governed by the diffusion coefficient (*D*), which for small organic molecules in common solvents typically falls in the range of 10^–9^ to 10^–10^ m^2^/s.^3,4^ At this rate, a molecule would require minutes to hours to traverse even a few centimeters, which might be a negligible limitation in small vials (such as HTE plates) and microchannels but a critical bottleneck in liter- or cubic-meter-scale reactors.

By contrast, convection involves bulk fluid motion, produced by magnetic stir bars, mixing elements, overhead impellers, or pumped circulation. Convection accelerates homogenization by continuously transporting material throughout the reactor, typically at rates orders of magnitude faster than diffusion. The effectiveness of convective mixing depends on multiple factors, including:

- Stirring speed and impeller geometry: Magnetic stir bars, overhead stirrers, static mixers, and baffles all generate different flow patterns, as indicated in Figure 1.^5–8^

- Reactor geometry and scale: The shape, size, and aspect ratio of vessels influence fluid dynamics and mixing times.^9,10^

- Physical properties of the reaction mixture: Viscosity, density differences, and interfacial tension affect mixing efficiency.^11^
- External fields: Variations in the magnetic field across a stir plate can create uneven stirring conditions, explaining why identical vials on the same plate may behave differently.^2^ Moreover, active mixing, for instance, through ultrasound, can be employed.^12^

**Figure 1:**
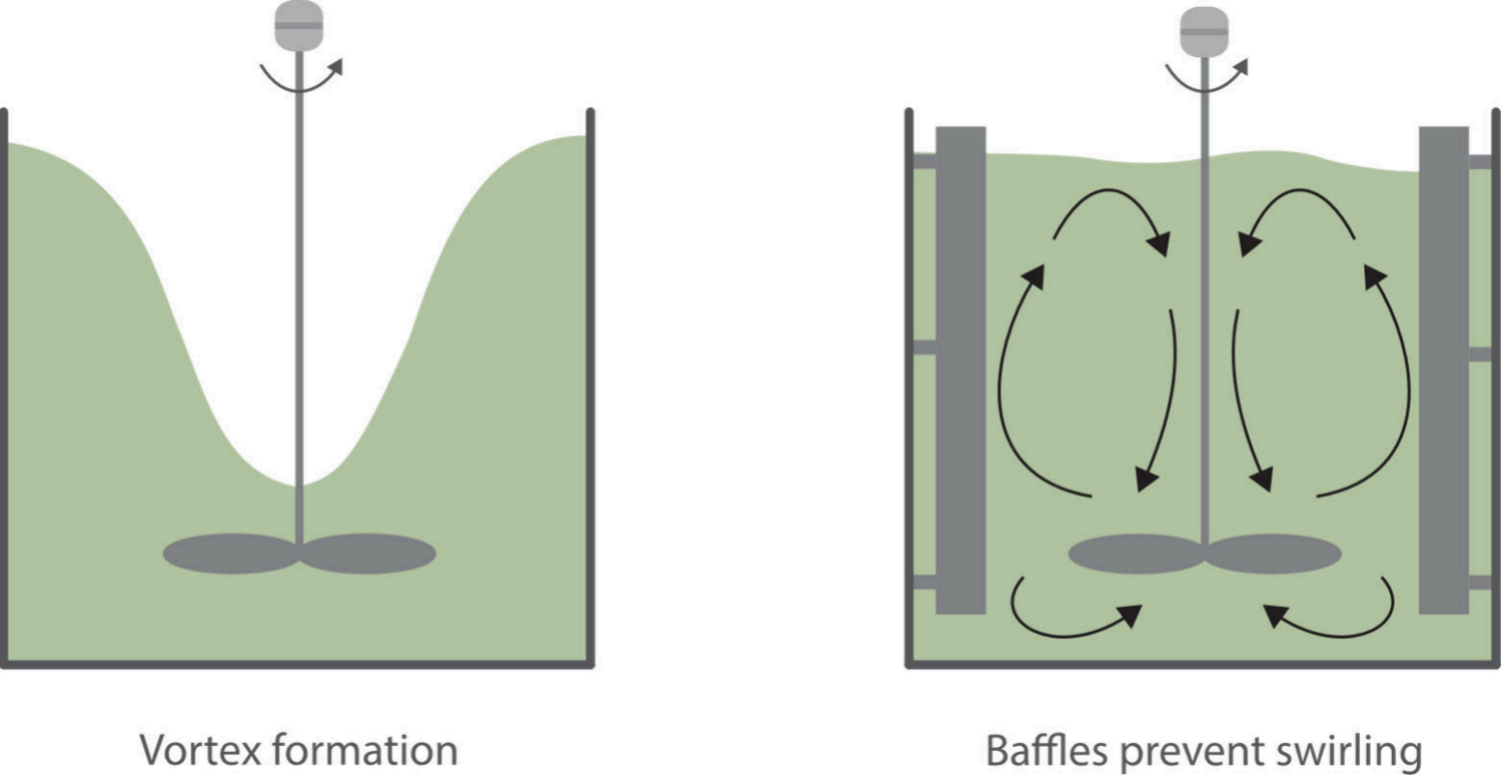
Without baffles (left), vortex formation occurs due to swirling fluid. Adding baffles (right) disrupts circular flow, promoting vertical mixing and enhancing overall mixing efficiency.

The **Sherwood number (Sh)** quantifies the interplay between convection and diffusion:

$$Sh=\frac{k}{D/L}$$

where *k* is the convective mass transfer coefficient, *L* is the characteristic length scale, and *D* is the diffusion coefficient. Large *Sh* values indicate convection dominates, while small values point to diffusion-dominated conditions. Correlations linking *Sh* to the Reynolds (*Re*) and Schmidt (*Sc*) numbers allow engineers to predict and optimize mass transfer for specific conditions.^13,14^

Let us consider two reacting species, **A** and **B**. The time required for them to encounter each other depends on both:

1. **How fast they move** (diffusion coefficients, convection velocity fields), and
2. **How far apart they are** (reactor volume, spatial distribution).

If only diffusion is present, increasing temperature boosts the diffusion coefficient, but this improvement might be insufficient. Reducing the reactor volume decreases diffusion distances and so lowers mixing times.^15^ This explains why microscale reactors often perform well without stirring. Indeed, specialized micromixer designs can reduce mixing times to the point where they outpace even extremely rapid rearrangement reactions.^16^ However, these findings do not extrapolate to pilot- or production-scale reactors (Figure 2), where diffusion limitations become more pronounced, and specialized mixer designs, often combined with baffles, are required to ensure adequate mixing.

**Figure 2:**
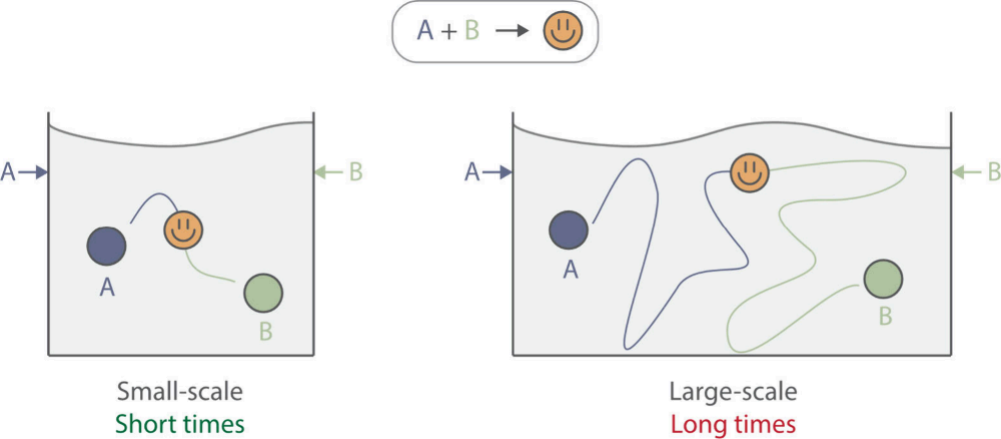
Illustration of mass transfer limitations during scale-up. At small scale, reactants A and B quickly encounter each other, enabling fast reactions. At large scale, distances are larger, increasing the time required for reactants to encounter each other and react.

Poor mixing can therefore lead to a range of issues, including localized concentration gradients, or “hotspots,” that promote side reactions or unwanted precipitation, zones where catalysts become saturated while the bulk of the solution remains underutilized, and inconsistent kinetics and yields when scaling a process from milligram-scale experiments to kilogram-scale production. Molecular diffusion alone may be sufficient in small-scale reactors with homogeneous and kinetically slow reactions that are premixed. However, as reactions increase in complexity, or thermal intensity, convective mixing becomes indispensable. Understanding and quantifying mixing through dimensionless numbers, scale-up principles, and hydrodynamic characterization is essential not only for achieving optimal yield but also for ensuring reproducibility, selectivity, and safety in synthetic chemistry.^17^

Mixing is not merely a convenience, it is often a prerequisite for ensuring reactants meet in both space and time.

## 3 Relevance to Organic Synthesis and Material Science

According to the Arrhenius equation, reaction rates depend on the frequency of effective molecular collisions. Without proper mixing, the number of collisions decreases, limiting reaction progress. Thus, mixing is not merely a convenience, it is often a prerequisite for ensuring reactants meet in both space and time.

In some cases, (vigorous) stirring does not improve reaction performance. This occurs when the rate-limiting step is governed by processes other than mass transfer. For example, if a reaction involves slow inherent kinetics, even perfectly mixed conditions will not accelerate the reaction (e.g., in photochemical transformations at low light intensities, the reaction can occur in a photon-limited regime and is therefore less sensitive to mass transfer).^18^ Here, diffusion alone may suffice, explaining the findings of Huang et al. for certain systems.^1,19^

This concept is well captured by the Damköhler number (*Da*), which relates the characteristic reaction time (τ_reaction_) to the characteristic mixing time (τ_mix_).^20^

$$Da=\frac{\tau_{mix}}{\tau_{reaction}}$$

In essence, if *Da* ≪ 1, the intrinsic reaction time greatly exceeds the mixing time scale, indicating that mixing has little to no impact on the observed yield. It is important to note, however, that in a vial with a 1 cm liquid height and typical molecular diffusion coefficients, the characteristic diffusion time is approximately 28 h. Thus, only very slow reactions would be fully homogenized by diffusion alone. It is important to note that this criterion assumes the reaction mixture starts homogeneous, with all reactants predissolved and no additional reagent feed during the reaction.

Temperature uniformity follows similar principles but is generally achieved much faster because thermal diffusivity is higher than molecular diffusivity. In addition, even small temperature differences typically induce natural convection in liquids, which greatly enhances heat transport beyond pure conduction. For water, the characteristic thermal equilibration time is approximately 700 s (about 12 min) for a 1 cm path length. In most small-scale laboratory experiments, this means temperature gradients are negligible as long as the system is well-heated or cooled, and losses to the environment are limited. However, for strongly exothermic reactions, heat generation can overwhelm passive dissipation, necessitating active cooling and vigorous stirring to prevent localized overheating and ensure safety.

It is important to note that identifying the rate-limiting step for a new reaction a priori is difficult. Because most reaction mechanisms involve multiple steps, including fast and slow equilibria, at least minimal stirring is recommended during methodology development to avoid unintentionally introducing hidden mass transfer limitations. In fact, it is recommended to perform experiments under two different stirring conditions (e.g., high and low) to confirm that the reaction is not limited by mass transfer (Figure 3). Conducting reactions in a mass-transfer-limited regime leads to distorted kinetics, which can in turn cause incorrect conclusions about intrinsic reaction rates, orders, or mechanistic behavior.^21,22^ Improving mixing can increase reaction rates, but this benefit must be weighed against the additional energy input or specialized equipment required for high-intensity mixing. At small laboratory scales, however, the incremental cost of stirring relative to static conditions is negligible, providing little justification for omitting agitation. Ultimately, the experimenter must decide on the appropriate balance between stirring intensity and the performance gains achieved, particularly when operating near a mass-transfer-limited regime.

**Figure 3:**
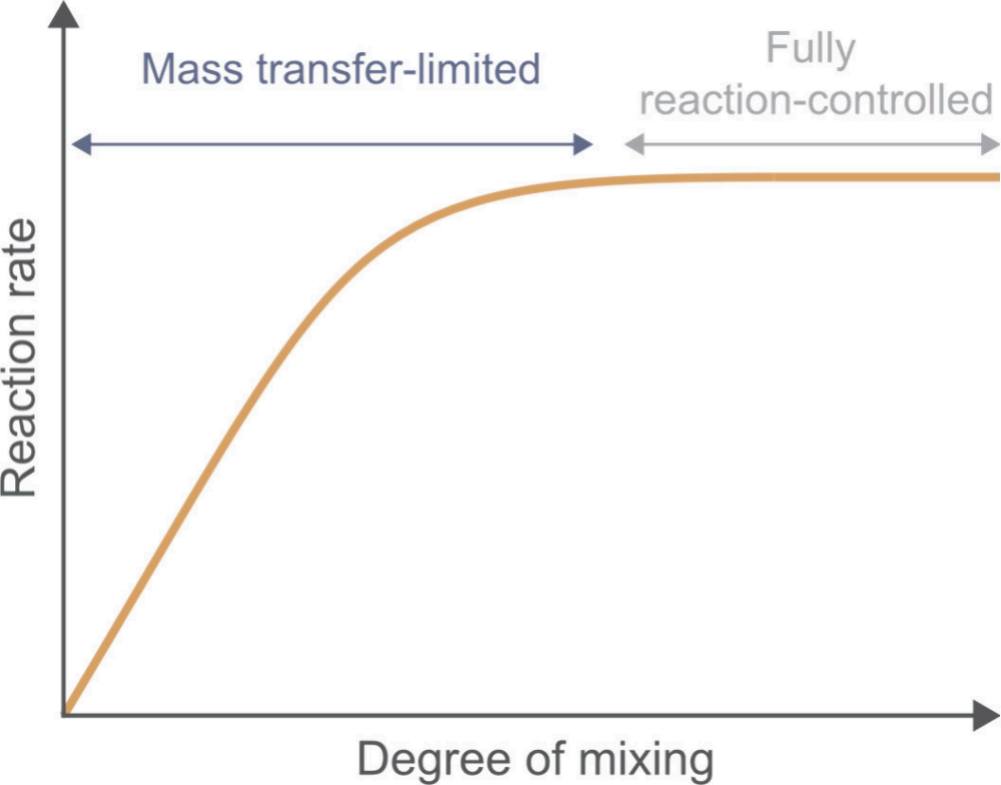
Dependence of the reaction rate on the degree of mixing. At low mixing levels, the process is mass transfer limited, and screening at different stirring conditions helps determine whether mass transfer influences the reaction rate.

At least minimal stirring is recommended during methodology development to avoid unintentionally introducing hidden mass transfer limitations.

Poor mixing can affect not only the overall reaction rate but also the selectivity of the transformation, as illustrated in Figure 4.^23,24^ Uneven concentration or temperature profiles within the vessel can favor undesired pathways. Examples include a reagent reacting preferentially with the product rather than the starting material, transient microenvironments leading to the loss of stereocontrol or regioselectivity, and product degradation occurring in localized hotspots. These problems become especially significant when the intrinsic reaction rate exceeds the characteristic mixing rate (*Da* > 1), a hallmark of mass transfer limitation that can give the false impression of altered chemical selectivity.^25^ Adequate stirring mitigates these effects by ensuring uniform reagent distribution and effective heat dissipation.

**Figure 4:**
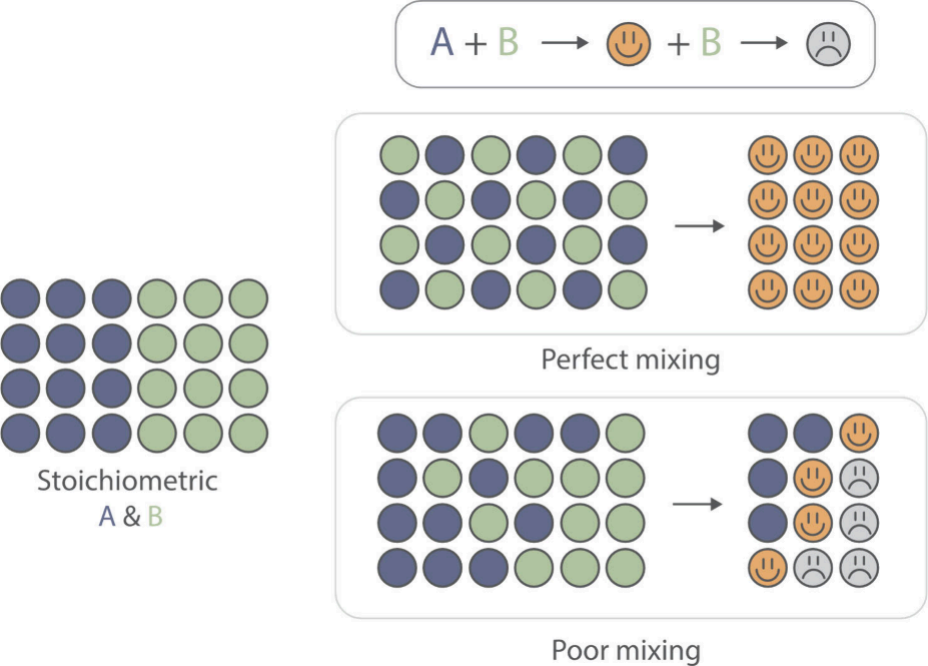
Poor mixing can cause local concentration gradients, leading to undesired side reactions and reduced selectivity. Under perfect mixing, reactants are uniformly distributed, favoring the desired product.

The importance of mixing is particularly evident in exothermic reactions, where inadequate agitation can create localized temperature spikes. Such thermal gradients not only accelerate unwanted side reactions but can also compromise stereochemical integrity or, in extreme cases, trigger thermal runaway, posing significant safety hazards. Even when the final isolated yield appears unaffected, these transient hotspots may still erode selectivity and reproducibility, highlighting why careful control of mixing is essential.

Basic mixing principles underlie why very fast reactions, such as lithium–halogen exchange, are typically carried out at dilute concentrations and under low-temperature conditions. For a simple first-order thermal reaction, the rate is directly proportional to both temperature and concentration, as described by the rate law:

$$r=kC=Ae^{-E_{a}/RT}C$$

By lowering the concentration (*C*) and temperature (*T*), the intrinsic reaction rate (*r*) decreases, allowing mixing to occur rapidly relative to the reaction itself. This ensures that the characteristic mixing time is shorter than the reaction time, enabling homogeneous conditions throughout the reactor (*Da* < 1). In other words, slowing the reaction in this way prevents localized concentration or temperature gradients from developing.

In recent years, microreactors and flow-based approaches such as flash chemistry have emerged as powerful tools to address these challenges.^26,27^ By dramatically reducing mixing times and enhancing heat transfer, these systems allow even very fast reactions to be conducted safely at higher temperatures and concentrations than would be possible in conventional batch reactors.^28^ In this context, the precise and rapid mixing achieved in microreactors ensures that reaction kinetics, rather than transport limitations, dictates the outcome.^29^

Poor mixing can affect not only the overall reaction rate but also the selectivity of the transformation.

Many important synthetic transformations are multiphase processes, such as gas–liquid, liquid–liquid, or gas–liquid–solid reactions.^30–34^ Each additional phase introduces extra resistance to the overall reaction, creating potential bottlenecks that limit reaction efficiency.^35,36^ In a typical gas–liquid–solid catalytic system, a gaseous reactant must first travel through the bulk gas phase, cross the gas–liquid interface, transfer through the liquid bulk, and finally reach the solid catalyst, penetrating its pores where the reaction takes place.^37,38^ In such systems, stirring can play a crucial role. Enhancing mixing can improve mass transfer rates, break bubbles and droplets into smaller units to significantly increase the available surface area, and keep solids suspended by maintaining a uniform distribution throughout the reactor.^39^ Our work, along with studies by others, has demonstrated that increasing rotor speed in photochemical oxidations markedly accelerates both gas–liquid and gas–liquid–solid processes.^40–43^ In one case study, the photochemical oxidation of α-terpinene to ascaridole using oxygen showed a strong dependence on rotor speed, with higher conversions observed at increased speeds.^43^ This improvement arises from enhanced bubble breakup and resulting in faster mass transfer, ultimately boosting reaction rates.

Examples where mixing directly influences both reaction rate and selectivity are abundant across synthetic chemistry and are therefore more the rule than the exception. Organolithium and Grignard reactions are highly sensitive to localized overheating, which can lead to safety hazards and undesired byproducts.^44,45^ Exothermic nitrations require efficient heat removal to prevent dangerous runaway reactions, while in polymerizations, chain length and molecular weight distribution are governed by the uniformity of mixing.^46–48^ Similarly, nanoparticle syntheses depend on precise control of nucleation and growth, and photochemical systems require the transport of species to irradiated zones.^49–51^ Electrochemical processes also rely on agitation to ensure consistent delivery of reactants to the electrodes.^52,53^ Notably, increasing rotation speeds has been observed to enhance yields and selectivities in the electrochemical methoxylation of *N*-formylpyrrolidine.^54^ These diverse examples illustrate how proper mixing can transform the performance of mass transfer-limited systems, and they highlight why entire families of reactor designs and impeller technologies have been developed specifically to meet these challenges.^10,55^

## 4 (Pre-)Mixing Practices

The way a reaction mixture is prepared and handled before the reaction begins can strongly influence the perceived role of mixing. Predissolving and homogenizing reagents can eliminate potential mass-transfer limitations that would otherwise arise during the reaction.^56^ Conversely, adding reagents directly into a static solution creates steep concentration and temperature gradients that require active agitation to overcome. This is why slow, controlled, dropwise additions are a common practice in organic chemistry: they limit local concentration spikes, allow heat to dissipate gradually, and help maintain selectivity.

Standardizing experimental protocols is therefore critical for reproducibility. Authors should explicitly report stirring conditions, vessel geometries, and addition techniques, as these can profoundly affect outcomes.^57^ Adjusting the mixing conditions offers a quick and effective means to determine if a reaction is mass-transfer limited and to assess whether increased agitation is necessary for optimal operation.

Mixing also plays a central role in process safety. Inadequate agitation can lead to localized hotspots, potentially causing solvent boiling, decomposition, or runaway reactions. At scale, insufficient mixing may result in temperature gradients, producing nonuniform reaction behavior and hidden hazards. Additionally, poor mixing can permit local accumulation of toxic side products or gases, posing risks of exposure or pressure build-up.

The importance of mixing is particularly evident in exothermic reactions, where inadequate agitation can create localized temperature spikes.

Qualitative assessments of mixing performance across different setups and operating conditions can be obtained using simple visualization methods such as dye-dilution tests. When more detailed quantitative information is required, several experimental and simulation-based tools are available. Competitive parallel reactions, most notably the Villermaux–Dushman system, are widely used to evaluate micromixing efficiency.^58–60^ In addition, residence-time-distribution studies and computational fluid dynamics (CFD) simulations provide further quantitative insights into mixing behavior over a wide range of reactor types and scales.^9,61–63^

For homogeneous systems, shifting from diffusion-controlled to convection-dominated regimes typically improves, or at least maintains, performance. However, multiphase systems are not always so forgiving; excessive stirring can sometimes induce unfavorable flow patterns like droplet coalescence, leading to poor mixing or reduced rates.^64^ In such cases, and ideally all cases, stirring speed must be carefully screened and optimized.

An important practical nuance is the effect of magnetic stirrer placement on reproducibility.^65^ Ananikov and co-workers revealed that even when conducting identical reactions side by side on the same stir plate, differences in vial position and height can significantly affect outcome.^2^ The authors showed that yield, reaction rate, and nanoparticle size distributions were all impacted by magnetic field variation across the plate surface. These reproducibility issues stem from inconsistent stir bar motion: some bars spun freely, others ground against vessel walls, and some stopped altogether. The study emphasized that for reliable results, vessels should be placed precisely at the center of the stir plate. This example highlights yet another layer of detail, which is often overlooked, where mixing control and experimental layout directly influence synthetic outcomes.

## 5 Conclusions

While recent studies have questioned the universal importance of stirring, such findings must be interpreted carefully and in context. For certain homogeneous reactions performed under ideal laboratory conditions, mass transfer may not be rate-limiting, leading to similar outcomes under both static and stirred conditions. However, as systems increase in complexity, involve multiple phases, or scale to larger volumes, mixing becomes a decisive factor influencing not only the reaction rate and selectivity but also safety and reproducibility.

Understanding the dynamic interplay between mass transfer and production rates is essential for both mechanistic investigations and the development of practical synthetic methodologies. Mixing is often treated as an afterthought, yet it is a fundamental variable that underpins the reliability of chemical data and the robustness of processes. Ultimately, thoughtful consideration of mixing effects should remain a cornerstone of both academic research and industrial process design, because, as the evidence overwhelmingly shows, mixing matters.

## Supplementary Material



## Notes

Transparent Peer Review report available (PDF)
