# Supplementary material for: Stirring the Debate: How Mixing Influences Reproducibility and Efficiency in Synthetic Organic Chemistry
Source: ACS Cent Sci. 2026 Jan 6;12(1):7–13. doi: 10.1021/acscentsci.5c01825 (PMC12871880; doi:10.1021/acscentsci.5c01825)
Supplement: Supplementary file 1 [file oc5c01825_si_001.pdf]

oc-2025-01825r.R1

Name: Peer Review Information for "Stirring the Debate: How Mixing Influences Reproducibility and Efficiency in Synthetic Organic Chemistry"

First Round of Reviewer Comments

Reviewer: 1

Comments to the Author

This manuscript by Noel, Schaaf and colleagues addresses the role of mixing (stirring) in organic synthesis, materials science, and process chemistry. The authors critically discuss recent reports suggesting that stirring may have limited influence on reaction outcomes. In contrast, they argue that mixing plays a crucial role in ensuring reproducibility, selectivity, safety, and scalability, particularly in heterogeneous systems and industrial settings.

The article is easy to read, well written and logically structured, and presents a well-balanced and technically sound perspective. The discussion is timely and relevant to both academic and industrial chemical research, and has value for a wide scientific audience, including students and early-career researchers who may underestimate the importance of mixing. I also appreciate the constructive and measured tone of the discussion.

I therefore recommend publication after addressing the following minor revisions:

- While the manuscript is intended as a commentary or perspective article, including one or two representative examples showcasing how mixing have affected yield, selectivity, or safety would reinforce discussion.

- Can the authors briefly comment on how other factors such as microwave irradiation, ultrasound (US), or the geometry of mixing elements (e.g., in flow systems) might influence mixing efficiency and reaction outcomes?

Reviewer: 2

Comments to the Author

1) The manuscript summarizes well-known concepts of diffusion, convection, and mixing parameters without providing new data, theoretical development, or quantitative analysis. It reads as a didactic overview rather than an original scientific contribution.

2) The discussion restates textbook principles without any experimental validation or modeling. Figures are schematic, qualitative, and do not present quantitative comparisons or insight.

3)The central message that “mixing always matters” is overstated and insufficiently supported. The authors fail to define clear boundaries or quantitative criteria where stirring influences or does not influence reaction outcomes.

4) Several sections overlap in content, and the article lacks a unifying framework or progression of ideas. The structure resembles an educational note rather than a focused scientific perspective.

5) There is no mention of automation, AI-controlled systems, or data-driven reproducibility—critical aspects of modern chemical research. This omission limits the paper’s relevance and innovation.

Reviewer: 3

Comments to the Author

This is an important and timely focus article on a fundamentally important question related to carrying out chemical reactions in solutions. The authors provide an accurate and balanced description. This article will be of great interest to professional chemists, as well as beginners and students. I enthusiastically recommend publishing the article.

One point to consider for revision is taking a closer look at catalytic conditions. Under stoichiometric reaction conditions, the reagents penetrate and contact each other in a somewhat more uniform environment.

In catalytic reactions with 0.01-0.0001 mol% catalyst, the reaction must ensure conditions in which a few catalyst molecules (typically large complexes with ligands or nanoparticles) should be in a contact with a large number of reacting molecules. With the conditions of sufficiently rapid renewing of near-catalyst local surrounding.

Do catalytic reactions require more detailed consideration of stirring? Are there nuances to ensure substrate-substrate versus substrate-catalyst spatial collisions?

Author's Response to Peer Review Comments:

Reviewer: 1

Recommendation: Publish in ACS Central Science after minor revisions noted.

Comments:
This manuscript by Noel, Schaaf and colleagues addresses the role of mixing (stirring) in organic synthesis, materials science, and process chemistry. The authors critically discuss recent reports suggesting that stirring may have limited influence on reaction outcomes. In contrast, they argue that mixing plays a crucial role in ensuring reproducibility, selectivity, safety, and scalability, particularly in heterogeneous systems and industrial settings.

The article is easy to read, well written and logically structured, and presents a well-balanced and technically sound perspective. The discussion is timely and relevant to both academic and industrial chemical research, and has value for a wide scientific audience, including students and early-career researchers who may underestimate the importance of mixing. I also appreciate the constructive and measured tone of the discussion.

We thank the reviewer for their comments on the manuscript.

I therefore recommend publication after addressing the following minor revisions:
- While the manuscript is intended as a commentary or perspective article, including one or two representative examples showcasing how mixing have affected yield, selectivity, or safety would reinforce discussion.

Answer: We highlighted relevant examples in the text of the manuscript.

- Can the authors briefly comment on how other factors such as microwave irradiation, ultrasound (US), or the geometry of mixing elements (e.g., in flow systems) might influence

mixing efficiency and reaction outcomes?

Answer: We have expanded the general discussion on mixing to include additional mixing-relevant elements, and we now also provide a brief discussion of active mixing approaches.

Reviewer: 2

We would like to thank the reviewer for their time to read the manuscript and provide their comments.

Recommendation: Does not meet the requirements of publishing in ACS Central Science.

Comments:
1) The manuscript summarizes well-known concepts of diffusion, convection, and mixing parameters without providing new data, theoretical development, or quantitative analysis. It reads as a didactic overview rather than an original scientific contribution.
2) The discussion restates textbook principles without any experimental validation or modeling. Figures are schematic, qualitative, and do not present quantitative comparisons or insight.
3)The central message that “mixing always matters” is overstated and insufficiently supported. The authors fail to define clear boundaries or quantitative criteria where stirring influences or does not influence reaction outcomes.
4) Several sections overlap in content, and the article lacks a unifying framework or progression of ideas. The structure resembles an educational note rather than a focused scientific perspective.
5) There is no mention of automation, AI-controlled systems, or data-driven reproducibility—critical aspects of modern chemical research. This omission limits the paper’s relevance and innovation.

Answer: We would like to clarify that this manuscript is intended as an In Focus article rather than an original research paper or a comprehensive review. Its purpose is to highlight a specific and often overlooked aspect of chemical practice, i.e. mixing, rather than to provide an exhaustive survey of the field. For readers seeking in-depth discussions, multiple detailed references are provided throughout the text.

With respect to the suggestion to include additional "hot topics" such as AI, automation, or data-driven reproducibility, we respectfully disagree that their omission limits the relevance of the piece. Expanding the scope to cover these broad and rapidly evolving areas would dilute the central message, which is to emphasize the foundational and persistent role of mixing in chemical reactions and reproducibility. We believe that maintaining focus serves the readership better and aligns with the purpose of the In Focus format.

That said, should the reviewer or editor feel that a brief contextual remark on these emerging areas would strengthen the article, we are happy to consider adding a short statement indicating how they intersect with mixing, without shifting the scope of the piece.

Reviewer: 3

Recommendation: Publish in ACS Central Science after minor revisions noted.

Comments:
This is an important and timely focus article on a fundamentally important question related to carrying out chemical reactions in solutions. The authors provide an accurate and balanced description. This article will be of great interest to professional chemists, as well as beginners and students. I enthusiastically recommend publishing the article.

One point to consider for revision is taking a closer look at catalytic conditions. Under stoichiometric reaction conditions, the reagents penetrate and contact each other in a somewhat more uniform environment.
In catalytic reactions with 0.01-0.0001 mol% catalyst, the reaction must ensure conditions in which a few catalyst molecules (typically large complexes with ligands or nanoparticles) should be in a contact with a large number of reacting molecules. With the conditions of sufficiently rapid renewing of near-catalyst local surrounding.
Do catalytic reactions require more detailed consideration of stirring? Are there nuances to ensure substrate-substrate versus substrate-catalyst spatial collisions?

Response: We thank the reviewer for this well-considered comment. Catalytic reactions indeed represent a special case in which the relative rates of the underlying processes become critical: for example, the intrinsic rate of the catalytic reaction, the rate of mass transport to the catalyst surface, or, in the case of photocatalysis, the lifetime of the excited catalytic species.

In the present manuscript, our intention is to remain general rather than focus on specific mechanistic cases. For that reason, we chose to use the overall reaction time as a consolidated parameter that implicitly captures these different contributions without introducing system-specific complexity.

Second Round of Reviewer Comments

Reviewer: 2

Comments to the Author

There are some recommendation

1) Clearly define at the outset what is genuinely new in this piece beyond existing reviews and tutorials on mixing, flow, and scale-up (e.g., a unifying framework for interpreting "stirring vs standing" results in synthetic methodology, or a decision tree for when stirring can be safely reduced).

2) Tighten the narrative so that every section explicitly supports that central contribution; consider removing or condensing general transport-phenomena material that does not directly change how synthetic chemists will design or interpret experiments.

3) Strengthen quantitative and comparative analysis

4) Where possible, move beyond qualitative statements ("mixing matters", "Da $\ll$ 1") by including simple, worked examples: typical values of Da, Sh, and characteristic times for representative reaction classes (e.g., fast organolithiums vs slow photochemistry vs multiphase oxidations).

5) Provide a more systematic comparison of the two highlighted 2025 studies on stirring vs standing, ideally in a small table (conditions, geometry, scales, mass-transfer regime), to make the reconciliation more concrete and instructive.

6) Currently, the most detailed examples are drawn from photochemical oxidations, rotor–stator spinning-disk reactors, flow systems, electrochemistry, and nanoparticle synthesis. Please broaden to include at least brief but concrete examples from other major synthetic

domains (e.g., catalytic C–C bond formation, organocatalysis, biocatalysis, polymerizations) where mixing has been quantified or shown to affect selectivity or safety.

7) Where literature exists, add references beyond the authors' own work to avoid the impression of a technology-centric rather than problem-centric perspective.

8) Suggested experimental checks for mass-transfer limitation (e.g., how much rate change vs stir-speed change is significant).

This would greatly increase the article's utility for non-engineering synthetic chemists, which is crucial.

Deepen the safety and reproducibility discussion

A small decision tree or schematic, distinguishing diffusion-dominated vs convection-dominated vs photon- or electron-limited regimes, would help readers rationally decide when "turning off the stirrer" is defensible

Author's Response to Peer Review Comments:

December 04, 2025

Re: Submission of Revised Version of our article "Stirring the Debate: How Mixing Influences Reproducibility and Efficiency in Synthetic Organic Chemistry" [Manuscript No. oc-2025-01825r.R1] to *ACS Central Science*

Dear Editor,

Thank you for the constructive feedback. In revising the manuscript, we have clarified the scope of the article, reinforced its accessibility for a broad chemistry audience, and added supporting references where appropriate. We maintained the educational focus of the piece by emphasizing general principles over system-specific details, while also highlighting practical diagnostics and safety considerations relevant to mixing in synthetic chemistry.

We would also like to note that Reviewers 1 and 3 in the previous round expressed strong support for publication, which reinforces that the article addresses an important and timely topic for the community. Attached you will find our detailed responses to Reviewer 2, as well as an annotated version of the manuscript in which all changes are highlighted.

We trust that the revisions address the concerns raised while preserving the intended purpose of an *In Focus* article: to provide a clear, concise, and broadly applicable overview of an essential concept in modern chemical practice.

Sincerely,

Tim Noel, on behalf of all coauthors.

Response to Reviewer 2

1) Clearly define at the outset what is genuinely new in this piece beyond existing reviews and tutorials on mixing, flow, and scale-up (e.g., a unifying framework for interpreting "stirring vs standing" results in synthetic methodology, or a decision tree for when stirring can be safely reduced).

Answer: Considering recent publications within the chemistry community, we saw the need for a digestible *In Focus* article that translates established engineering principles into practical guidance for synthetic chemists; there is simply nothing comparable currently available in the chemical literature. Our aim is to make these concepts accessible to a broad audience by combining our own observations with well-described transport phenomena and relevant illustrative examples.

In our view, the core message is straightforward: do not leave reactions standing. From a safety perspective, it is risky to forgo stirring in exothermic reactions, where localized heat accumulation can lead to hazardous situations. In addition, there is little reason to accept the possibility of operating in a mass-transfer-limited regime when minimal stirring effort can substantially reduce that risk.

A simple and unifying framework consists of varying the stirring conditions (we recommend testing two stir rates), optionally measuring temperature, and reporting experimental details meticulously, such as order of addition and whether reagents were premixed. We have emphasized these practical considerations throughout the manuscript.

2) Tighten the narrative so that every section explicitly supports that central contribution; consider removing or condensing general transport-phenomena material that does not directly change how synthetic chemists will design or interpret experiments.

Answer: The relevance of mixing to synthetic methodology directly follows from these underlying transport principles, which is why we chose to elaborate on them in a digestible, accessible way. Our aim is to highlight their importance for synthetic chemists (and related communities) without overwhelming readers with overly complex equations. Experience shows that extensive mathematical detail can discourage engagement, especially from audiences not routinely trained in chemical engineering. Precisely because many synthetic chemists do not routinely consider these general mass- and heat-transfer phenomena, it is essential to bring them to the forefront. These principles form the foundation of any chemical process, and overlooking them can easily lead to misinterpretation of reaction kinetics, perceived selectivity effects, or even unsafe operating conditions.

3) Strengthen quantitative and comparative analysis

Answer: We elaborated the part on comparative, qualitative, and quantitative judgement.

4) Where possible, move beyond qualitative statements (“mixing matters”, “Da $\ll$ 1”) by including simple, worked examples: typical values of Da, Sh, and characteristic times for representative reaction classes (e.g., fast organolithiums vs slow photochemistry vs multiphase oxidations).

Answer: For an *In Focus* article, our goal is to provide a general, easy-to-consult resource that is applicable across many reaction systems. In all cases, the balance between mixing and reaction rate is critical, which is why we reiterate the importance of the Damköhler number (Da), a well-established concept in the literature. However, the distinct numerical values for mixing time and reaction time are not always readily available for a given setup.
Although specific examples do exist, such as those reported by Jensen[1], they are highly system-dependent and would shift attention away from the main message. Moreover, including numerical examples risks misleading generalizations, as both Da and Sh (Sherwood number) depend strongly on reactor geometry, stirrer type, and flow regime. For these reasons, we chose to present the governing principles and direct readers to specialized engineering literature where system-specific correlations are provided.

1) Nagy, K. D.; Shen, B.; Jamison, T. F.; Jensen, K. F. Mixing and Dispersion in Small-Scale Flow Systems. Org. Process Res. Dev. 2012, 16 (5), 976–981. https://doi.org/10.1021/op200349f.

5) Provide a more systematic comparison of the two highlighted 2025 studies on stirring vs standing, ideally in a small table (conditions, geometry, scales, mass-transfer regime), to make the reconciliation more concrete and instructive.

Answer: The two highlighted studies served primarily as inspiration to discuss the underlying engineering principles of mixing; they are not meant to be central to the work itself. We refer to them only to illustrate that misconceptions exist within the community. Delving into their specific details would not enhance the general understanding we aim to provide, nor would it help convey our broader message.
A comparative table of the two studies would similarly shift the focus away from the fundamental principles toward case-specific nuances. Our goal is not to critique individual papers, but to demonstrate that such divergent conclusions naturally arise when mass- and heat-transfer regimes differ. Emphasizing the general principles therefore provides a more robust and broadly applicable framework for the readership.

6) Currently, the most detailed examples are drawn from photochemical oxidations, rotor–stator spinning-disk reactors, flow systems, electrochemistry, and nanoparticle synthesis. Please broaden to include at least brief but concrete examples from other major synthetic domains (e.g., catalytic C–C bond formation, organocatalysis, biocatalysis, polymerizations) where mixing has been quantified or shown to affect selectivity or safety.

Answer: We deliberately included a broad range of reaction classes in the article, extending from extremely exothermic transformations (such as nitrations) to polymerizations, to illustrate how mixing considerations manifest across diverse chemistries. These examples are already discussed within the work and serve to emphasize the wide applicability of the principles we outline.

7) Where literature exists, add references beyond the authors' own work to avoid the impression of a technology-centric rather than problem-centric perspective.

Answer: Providing a comprehensive literature list would be challenging considering that the first reports date back to the 1930s. We try to highlight our own experiences, while at the same time refer to the pioneers and other relevant work. To further complete the literature list we have added additional references to the manuscript (13 references added).

8) Suggested experimental checks for mass-transfer limitation (e.g., how much rate change vs stir-speed change is significant). This would greatly increase the article's utility for non-engineering synthetic chemists, which is crucial.

Answer: In theory, any change in stirrer speed will affect a reaction operating in a mass-transfer-limited regime, although whether this difference is experimentally observable depends on the magnitude of the effect relative to the experimental error. For most chemists, a simple stir-speed test already provides a useful qualitative diagnostic: if the reaction rate changes with rpm, the system is mass-transfer limited. We now emphasize this practical check more explicitly in the text.
Whether the observed change in reaction rate is considered significant is necessarily at the discretion of the experimenter and depends on practical factors such as analytical resolution and available equipment. The fact that improving mass-transfer properties can influence reaction outcomes has been demonstrated clearly by Schouten and co-workers[1] (k_La refers to the volumetric interfacial mass transfer coefficient), as well as many others (additional references have been included in the manuscript).

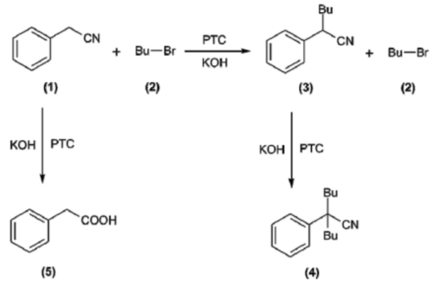

Table 1. Organic Slug Lengths and Specific Interfacial Surface Areas at Different Volumetric Aqueous-to-Organic Phase Flow (AO) Ratios[a]

aqueous-to-organic phase volumetric flow ratio	average organic slug length (μm)	average surface-to-volume ratio (m^2/m^3)	k_La ($m_i^3/(m_r^3$ s))	conversion at 80 °C and 9.8-min residence time (%)
1.0	467	3000	0.24	40
2.3	330	4500	0.36	74
4.0	295	5100	0.41	92
6.1	265	5900	0.47	99

1) Jovanović, J.; Rebrov, E. V.; Nijhuis, T. A.; Hessel, V.; Schouten, J. C. Phase-Transfer Catalysis in Segmented Flow in a Microchannel: Fluidic Control of Selectivity and Productivity. Ind. Eng. Chem. Res. 2010, 49 (6), 2681–2687. https://doi.org/10.1021/ie9017918.

Deepen the safety and reproducibility discussion A small decision tree or schematic, distinguishing diffusion-dominated vs convection-dominated vs photon- or electron-limited regimes, would help readers rationally decide when "turning off the stirrer" is defensible

Answer: Our intention is to remain general and to compare relative rates rather than delve into situation-specific details. At no point do we recommend turning off stirrers when possible. On the contrary, from a safety perspective this would be ill-advised. We also emphasize the trade-off

between increased stirring intensity and the associated energy or equipment costs, particularly at larger scales. At small scales, minimal stirring already mitigates most risks effectively (e.g., magnetic stirring in mL-scale reactors). However, from an engineering safety standpoint, active mixing is the default precaution. Diffusion alone cannot ensure homogeneity under exothermic conditions or during scale-up, making reliable stirring essential for safe and reproducible operation.
